# Decellularization of placentas: establishing a protocol

**DOI:** 10.1590/1414-431X20176382

**Published:** 2017-11-17

**Authors:** L.C.P.C. Leonel, C.M.F.C. Miranda, T.M. Coelho, G.A.S. Ferreira, R.R. Caãada, M.A. Miglino, S.E. Lobo

**Affiliations:** 1 Setor de Anatomia, Departamento de Cirurgia, Faculdade de Medicina Veterinária e Zootecnia, Universidade de São Paulo, São Paulo, SP, Brasil; 2 Universidade Metodista de São Paulo, São Paulo, SP, Brasil; 3 Universidade São Judas Tadeu, São Paulo, SP, Brasil

**Keywords:** Extracellular matrix, Placenta, Decellularization, Acellular biomaterial

## Abstract

Biological biomaterials for tissue engineering purposes can be produced through tissue and/or organ decellularization. The remaining extracellular matrix (ECM) must be acellular and preserve its proteins and physical features. Placentas are organs of great interest because they are discarded after birth and present large amounts of ECM. Protocols for decellularization are tissue-specific and have not been established for canine placentas yet. This study aimed at analyzing a favorable method for decellularization of maternal and fetal portions of canine placentas. Canine placentas were subjected to ten preliminary tests to analyze the efficacy of parameters such as the type of detergents, freezing temperatures and perfusion. Two protocols were chosen for further analyses using histology, scanning electron microscopy, immunofluorescence and DNA quantification. Sodium dodecyl sulfate (SDS) was the most effective detergent for cell removal. Freezing placentas before decellularization required longer periods of incubation in different detergents. Both perfusion and immersion methods were capable of removing cells. Placentas decellularized using Protocol I (1% SDS, 5 mM EDTA, 50 mM TRIS, and 0.5% antibiotic) preserved the ECM structure better, but Protocol I was less efficient to remove cells and DNA content from the ECM than Protocol II (1% SDS, 5 mM EDTA, 0.05% trypsin, and 0.5% antibiotic).

## Introduction

Extracellular matrices (ECM) of decellularized tissues and/or organs have been widely used as biological biomaterials for tissue engineering purposes (1–3). Decellularization protocols are tissue/organ specific and can be performed using chemical, physical and/or enzymatic methods (2,4).

An optimal decellularization protocol should guarantee complete cell and nucleic acids removal, preserve ECM physical structure and chemical composition (1,2) and cause no immune reactions in the host organism (5,6). However, decellularization processes inevitably affect ECM at different levels; therefore, distinct protocols must be tested and established for each tissue/organ of interest, where variables such as tissue thickness, ECM and cell density must be considered (5,7). Once decellularization is achieved, the size, shape, composition and mechanical properties of acellular matrices may influence the indication for potential clinical applications (8). Decellularization has been tested in several tissues and organs such as murine hearts (9), murine and human lungs (10), porcine kidneys (11) and in different types of placentas (12,13).

Placentas are organs rich in ECM and are an important source of stem cells (14–16). Placentas are composed of fetal and maternal portions, which are localized closer to the fetus and to the uterus, respectively, and can be classified according to distinct parameters, such as gross anatomy, type of fetal-maternal interface and fetal maternal interdigitation (12,13,17).

ECM from decellularized human placentas have been clinically applied for soft tissue reconstruction (18), such as for the treatment of patients subjected to lumpectomy (12). It has also been demonstrated that decellularized placentas have matrix components similar to those found in skin, along with growth factors and bioactive molecules involved in dermal wound healing (19). Additionally, placental ECM, including the chorionic plate, allows adhesion and proliferation of cells like those derived from adipose tissues, epithelium and keratinocytes (19,20).

Placentas are commonly discarded after birth (16); thus, they do not require an invasive procedure to be harvested, which is an important advantage over other tissues and organs (17,18). Placentas are abundant in volume and can be fragmented in different shapes, size and thickness (12,20). Their ECM has been shown to provide mechanical strength to support tissue development and neovascularization; both maternal and fetal portions can be degraded after implantation and favor tissue remodeling and regeneration (12).

Biological biomaterials, similar to those composed of ECM from decellularized placentas, are classified according to the origin into xenografts (the tissue donor and the receptor are from different species), allografts (the tissue donor and the receptor are different individuals from the same species) or autografts (the tissue donor and the receptor are the same individual). The possibility of causing immunological reactions, the total volume of tissue available and the transmission of pathogens must be taken into account, when choosing the best tissue origin and source (20).

Decellularized placentas may be used as xeno-, allo-, and even as autografts, comprising a broad spectrum of potential applications. Therefore, this study aimed at comparing the efficacy of protocols to decellularize fetal and maternal portions of canine placentas, which represents the first step for future investigations, particularly in veterinary medicine.

## Material and Methods

### Canine placentas and ethical approval

This study was approved by the Ethics Committee in Animal Experimentation of the Faculdade de Medicina Veterinária e Zootecnia, Universidade de São Paulo (CEUA/FMVZ-USP). Canine uteruses from pregnant dogs were harvested in castration campaigns after ovariosalpingohisterectomy procedures. The gestational age of the fetuses was defined using the crown-rump measurement, which is based on the distance between the highest point of the fetal head and the most proximal point of the tail, as describe by Evans and Sack (21).

### Decellularization methods

Placentas were initially dissected, washed in water and separated into the maternal and fetal portions.

In the first part of the study, ten different decellularization protocols were tested (Table 1). These varied according to: 1) types of detergents (ionic and anionic) and other reagents used for cell removal, 2) utilization of immersion and/or perfusion methods, and 3) freezing temperatures before decellularization. Among these protocols, two were selected for further analyses, based upon the following criteria: 1) tissue transparence (the higher the translucency the better), and 2) histological analysis, used to identify remaining cells in ECM. The protocols that led to placental samples with no translucent aspect or with remaining cells were considered ineffective.

**Table 1.** Description of 10 protocols for placental decellularization and their variables: reagents, duration of the process, number of placentas (fetal and maternal portions) and gestational ages of the fetuses (crown rump).

Temperature	Reagents	Duration	Fetal portion	Maternal portion	Crown rump
Protocol I - Immersion					
4°C	1% SDS or SDS+10 mM TRIS	2 days	8	8	40 days
	1% Triton X-100	2 days			
4°C	1% SDS or SDS+10 mM TRIS	3 days	8	8	
	1% Triton X-100	2 days			
Protocol II - Immersion					
-20°C or -80°C	SDS+10 mM TRIS	10 days	20	4	47 days
	1% Triton X-100	2 days			
Protocol III - Immersion					
-20°C or -80°C	SDS+10 mM TRIS	10 days	6	1	50 days
	1% Triton X-100	2 days			
Protocol IV - Perfusion					
-20°C or -80°C	SDS+10 mM TRIS	10 days	2 (whole placentas)	-	
	1% Triton X-100	2 days			
Protocol V - Immersion					
Room temperature	SDS+10 mM TRIS	9 days	6	4	53 days
	1% Triton X-100	2 days			
Protocol VI - Perfusion					
Room temperature	SDS+10 mM TRIS	8 days	1 (whole placenta)	-	
	1% Triton X-100	2 days			
Protocol VII - Immersion					
Room temperature	SDS+10 mM TRIS	3 days	12	7	43 days
	1% Triton X-100	2 days			
Protocol VIII - Perfusion					
Room temperature	SDS+10 mM TRIS	8 days	1 (whole placenta)	-	
Protocol IX - Immersion					
Room temperature	1% SDS	4 days	4	4	55 days
	5 mM EDTA+0.5% ATB or 50 mM TRIS+5 mM EDTA+0.5% ATB	2 days			
	DNAse-I	5 h			
	70% ethanol	1 h 30 min			
Protocol X - Immersion					
Room temperature	1% SDS	4 days	6	6	56 days
	5 mM EDTA+0.05% trypsin+0.5% ATB or 50 mM TRIS+5 mM EDTA+0.5% ATB	2 days			
	70% ethanol	1 h 30 min			

SDS: sodium dodecyl sulfate; EDTA: ethylenediamine tetraacetic acid; ATB: antibiotic (penicillin-streptomycin).

Two detergents were used to remove cells: ionic sodium dodecyl sulfate (SDS) and non-ionic Triton X-100. Both were tested at 1% concentration, as previously described (12,18). Other reagents were associated to improve the decellularization process: the calcium chelator EDTA and the enzymatic agent trypsin.

Eighty-six samples of fetal portions (that are thicker than the maternal) and fifty-five of maternal portions from different canine placentas were immersed (and not perfused) in the reagents (immersion method). Both portions were then washed with water, sectioned with approximately 2.5×2.5 cm and immersed in decellularization solutions. Samples kept in immersion were also maintained in agitation using the Agitador TS-2000A type VDRL Shaker (Biomixer, USA), during the day. Four samples of fetal portions were subjected to perfusion method. For this procedure, samples were initially washed with water, cannulated and perfused for approximately 12 h a day (protocols 4, 6, and 8; Table 1) using JYM Infusion Pump JSB-1200 (Jian Yuan Medical Technology Co. Ltd., China), with a flow rate of 150 mL/h.

Two different freezing temperatures, to which samples were exposed before the beginning of the decellularization, were tested: –20°C (applied to 14 fetal portions and 1 maternal portion) and –80°C (applied to 16 fetal and 4 maternal portions). Incubation with agitation, at room temperature, was used for 49 samples (i.e., 29 from fetal and 21 from maternal portions; Protocols 5 to 10; Table 1).

Based on these 10 protocols, two (Table 2) were chosen based on the criteria previously described. The selected protocols differed from each other regarding the utilization of EDTA+Tris *vs* EDTA+trypsin. The other parameters, i.e., time of incubation in SDS and Triton X-100 (detergents), antibiotic solution (penicillin-streptomycin) and washes (including ethanol 70%) remained unaltered.

**Table 2.** Distribution of fetal and maternal samples into the two selected placenta decellularization protocols.

ID	CR	Fetal portion		Maternal portion	
		Protocol I	Protocol II	Protocol I	Protocol II
Animal 1	54	2	2	2	1
Animal 2	55	2	2	2	2
Animal 3	54	2	2	2	2
Animal 4	51	2	2	2	2
Total samples		8	8	8	7

ID: identification; CR: crown rump length (mm); Protocol I: [1% SDS, 5 mM EDTA, 50 mM TRIS, and 0.5% antibiotic]; Protocol II [1% SDS, 5 mM EDTA, 0.05% trypsin, and 0.5% antibiotic].

Briefly, fetal portions were cannulated and perfused with water until complete drainage of blood from the vessels (approximately 200 mL). Both portions (fetal and maternal) were fragmented (2×2 cm), washed 3 times with distilled water for 5 min and incubated with 1% SDS for 4 days (3 changes on the first day and 2 on the others). This was followed by 3 washes of 5 min with buffer solution (40 mM HEPES, containing penicillin-streptomycin 0.5%). At this point, samples were divided and immersed in two different decellularization solutions: Protocol I (5 mM EDTA, 50 mM TRIS, and 0.5% antibiotic) or Protocol II (5 mM EDTA, 0.05% trypsin, and 0.5% antibiotic). These solutions were changed twice a day and samples remained immersed for 48 h. For both protocols, the next steps corresponded to 3 washes of 5 min with buffer solution (40 mM HEPES + 0.5% antibiotic) and immersion in 1% Triton X-100 for 2 days (changed twice a day). Protocols ended with 3 washes in a solution containing PBS 1X and 0.5% antibiotic (for 1 h each) followed by 3 washes with 70% ethanol for 30 min and 3 washes with PBS 1X combined with 0.5% antibiotic (30 min). Throughout the decellularization process, samples were maintained in agitation at room temperature during the day and at 4°C, without agitation, overnight.

Protocol I was tested in 8 fetal portions of placentas from 4 different bitches (1 fragment harvested from 2 different placentas, from each pregnant dog) and in 8 maternal portions as described above, totalizing 16 samples. Protocol II, was also tested in 8 fetal and 7 maternal portions, as described above, totalizing 15 samples (Table 2).

### Histological analyses

Histological analyses were performed in decellularized samples and in control groups (non-decellularized samples). The fetal and maternal portions were fixed in 4% paraformaldehyde, dehydrated with increasing concentrations of alcohol (70, 80, 90, and 100%), diaphanized in xylene and included in paraffin. Samples were sectioned in 5-µm thickness, stained with hematoxylin-eosin (HE), Masson's trichrome and Picrosirius red and analyzed under light microscope Nikon 80i (Nikon, Japan) and microscope Carl Zeiss (Zeiss, Germany).

### Scanning electron microscopy

Decellularized samples were fixed in 4% paraformaldehyde, washed with distilled water in ultrasound (2 washes for 2 min and 4 washes for 5 min) and maintained in 70% ethanol overnight. Dehydration was performed using ethanol (80 and 90% for 5 min, followed by 100% ethanol, 3 times for 10 min each). The critical point drying was conducted using CPD020 Balzers Union, followed by the gold coating and analysis using Scanning Electron Microscope model Leo 435 VP' (Zeiss, Germany).

### DNA content assay

DNA content was assessed in samples decellularized according to Protocols I (8 samples from each portion, i.e. fetal and maternal) and II (7 samples per portion), using the Quant-iT^TM^ PicoGreen^®^ dsDNA reagent assay (Life Technologies, USA), following the manufacturer's recommendations.

Briefly, decellularized samples were sectioned in 3×3 mm sections and placed on 96 wells plate; 100 µL of PicoGreen^®^ solution was added to each well and samples were incubated for 18 h, at 37°C. The supernatants were transferred to black plates (Falcon^®^, USA), read in spectrophotometer at wavelength of 480 nm of excitation and 520 nm of emission and analyzed using SoftMax Pro6 Program (SpectraMax^®^Paradigm^®^, USA). The experiment was performed in triplicate.

### Immunofluorescence

Samples from fetal and maternal portions of control and decellularized placentas (Protocols I and II) were immersed in Optimal Cutting Temperature (OCT), frozen in liquid nitrogen and maintained at –150°C. Samples were sectioned with 8 µm of thickness, maintained at room temperature for 1 h and fixed with cold acetone for 10 min at 20°C. Slices were dried at room temperature for 20 min, incubated with 2% tris-buffered saline/bovine serum albumin solution (TBS/BSA) for 1 h and incubated with primary antibody overnight at 4°C in humidified chamber. Slices were then washed 3 times for 5 min with 0.2% TBS/BSA solution in room temperature and incubated with second antibody (1:300) for 1 h. Three other washes, 5 min each, followed by washes with 0.2% TBS/BSA solution were performed. Slices were incubated with DAPI (1:10.000) for 10 min at room temperature, washed 3 times with TBS 1x (5 min each), sealed with glycerol/PBS 1x (1:1) and nail polish, and analyzed in confocal microscopy in FV1000 Olympus IX81 (Japan) with a 400× objective, in five different fields.

Primary antibodies corresponded to: anti-laminin (ab11575 - ABCAM, USA), anti-fibronectin (ab2413, ABCAM), anti-collagen type I (25974, Santa Cruz, USA) and anti-collagen type III (sc8779, Santa Cruz; dilution of 1:200). Secondary antibodies were: Alexa Fluor^®^ 488 goat anti-Rabbit (A-11008, Life Technologies) or Alexa Fluor^®^ 488 rabbit anti-Goat (A-11078, Life Technologies).

### Statistical analysis

Data of DNA quantification was analyzed with Pearson correlation, which divides the covariance of the variables by their respective standard deviations. Therefore, linear regression was calculated to determine DNA concentration based on fluorescence values. Afterwards, a study of cross correlation between the values of both portions of the placenta (maternal and fetal) and both protocols (I and II) was performed.

## Results

### Analyses of variables from ten preliminary protocols

#### Association of different decellularization solutions

**Figure 1.**
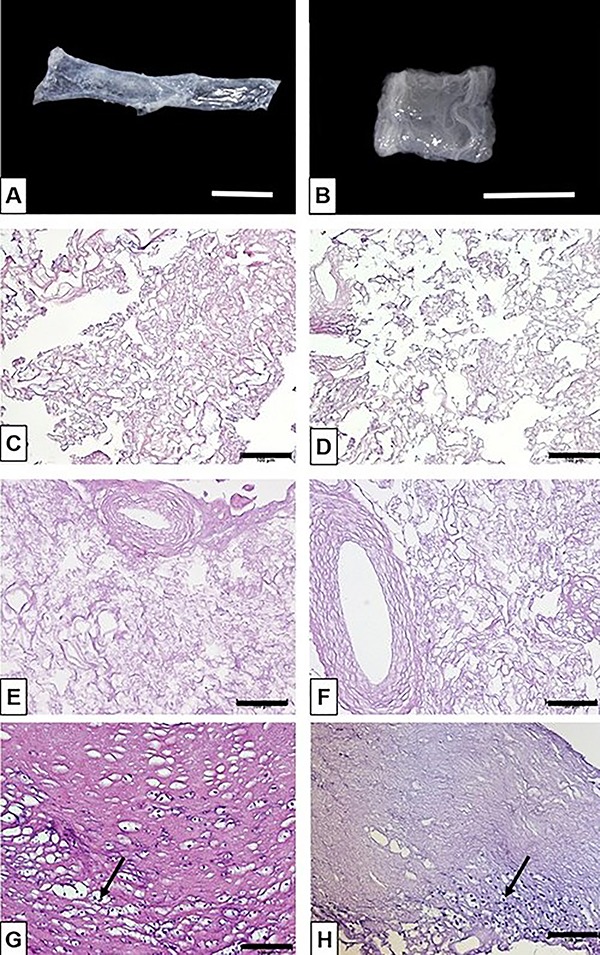
Macroscopic and microscopic aspects of fetal (*A–F*) and maternal (*G* and *H*) portions of canine placenta subjected to decellularization. Perfusion (*A, C, E* and F) and immersion (*B, D*) techniques in the fetal portion of placenta led to cell removal and samples with gelatinous and translucent aspects. No apparent differences regarding the percentage of remaining cells were found in placentas frozen at –20°C (*C, E*) and –80°C (*D, F*) prior to decellularization. Perfused placentas did not decrease the incubation time and frozen placentas required more time of incubation with decellularization reagents to remove cells. The application of 1% sodium dodecyl sulfate detergent (*G*) and its association with 1% Triton X-100 (*H*) were ineffective to completely remove cells (black arrows) from the maternal portion of placenta. *A* and *B*: Scale bar = 1 cm. *C*-*H*: HE staining; 20×; scale bar = 100 µm.

The utilization of different detergents (SDS and Triton X-100) led to samples with gelatinous aspect and preservation of blood vessels (Figure 1A and B). The application of 1% SDS alone did not remove cell content after four days of incubation, when it was observed cell nuclei in histological sections (Figure 1G). Similar findings were observed with the association of 1% SDS + 1% Triton X-100 (Figure 1H) in the maternal portion of canine placenta. Therefore, other reagents, such as EDTA, Tris and trypsin were added to the protocols to improve cell removal.

#### Immersion with or without perfusion

Perfusion of placentas with water before the utilization of detergents aimed at removing blood from the vessels. It was performed only on fetal portions due to their increased thickness and blood vessels network. Perfused samples (Figure 1A, C, E, F) showed similar results compared to samples subjected to immersion method only (Figure 1B and D), with respect to translucence (macroscopy) and cell removal (microscopy). Therefore, both methods (immersion/perfusion) promoted cell removal (Figure 1C and D), although the perfusion used in this study did not decrease the time required for tissue decellularization. Considering the time needed (similar for both methods) and the volume of decellularization reagents (higher with perfusion), immersion was chosen for further decellularization protocols.

#### Temperature gradients

Placentas frozen at -20°C (Figure 1C and E) and -80°C (Figure 1D and F) before decellularization were compared to samples kept at 4°C. Frozen samples that were not previously washed with water had to remain longer periods of time immersed in detergent solutions to acquire transparency, which may have contributed to a greater disruption of the ECM as observed in the histological analysis (Figure 1D). Conversely, freezing tissues before exposure to detergents improved cell removal (Figure 1C, E, F).

### Analyses of Protocols I and II

#### Macroscopic analysis

Protocols I (5 mM EDTA; 50 mM TRIS; 0.5% antibiotic) and II (5 mM EDTA + 0.05% trypsin + 0.5% antibiotic) were tested in both portions (fetal and maternal) of the placentas.

**Figure 2.**
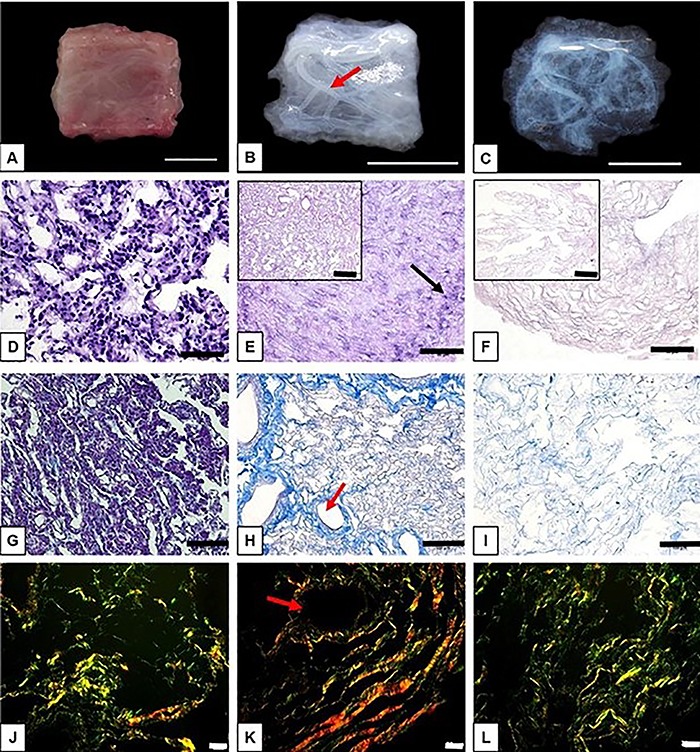
Macroscopic and microscopic aspects of fetal portions of canine placenta. *Left column* (control group), *middle column* (Protocol I – 5 mM EDTA + 50 mM TRIS + 0.5% antibiotic) and *right column* (Protocol II – 5 mM EDTA + 0.05% trypsin + 0.5% antibiotic). Protocol I led to better conservation of the macroscopic and microscopic structures. In *E* (upper left corner, 20×; scale bar = 100 µm) it is possible to observe a structurally organized extracellular matrix (ECM), but with nuclei of remaining cells (black arrow). Vascular wall architecture (red arrow) was preserved (*B*, *H*, and *K*). Protocol II was more aggressive to ECM as shown in *F* (upper left corner, 20×; scale bar = 100 µm), but was more effective in removing cells. *A*-*C*: macroscopic aspect, scale bar = 1cm; *D-F*: HE staining, 40×, scale bar = 50 µm; *G-*I*:* Masson's trichrome staining, 20×, scale bar =100 µm; *J-L*: fibrillar collagen network under polarized light, Picrosirius red staining, objective 16, magnification 1.25, scale bar = 20 µm.

**Figure 3.**
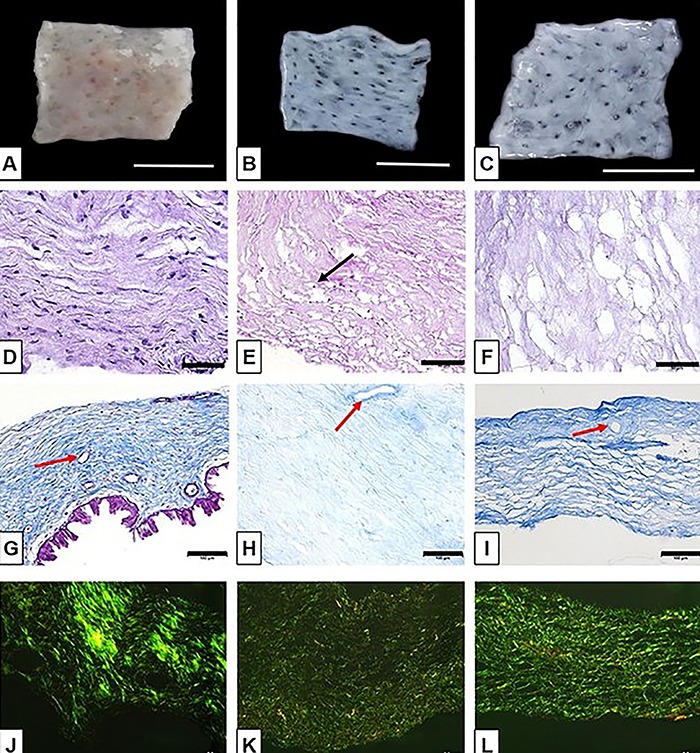
Macroscopic and microscopic aspects of maternal portions of canine placenta. *Left column* (control group), *middle column* (Protocol I - 5 mM EDTA + 50 mM TRIS + 0.5% antibiotic) and *right column* (Protocol II - 5 mM EDTA + 0.05% trypsin + 0.5% antibiotic). Macroscopic changes were not observed when Protocols I (*B*) and II (*C*) were compared. In Protocol I (*E*) cell content was not eliminated completely (black arrow). Samples subjected to Protocol II showed less cells and ECM derangement (*F*). Blood vessels structure (red arrow) were more preserved in Protocol I (*H*). *A–C*, macroscopic aspect, scale bar = 1 cm; *D–F*, HE staining, 40×, scale bar = 50 µm; *G*–*I*, Masson's trichrome staining, 20×, scale bar = 100 µm; *J*–*L*, fibrillar collagen network under polarized light, Picrosirius red staining; objective 16; magnification 1.25; scale bar = 20 µm.

Samples prepared according to Protocol II were fragile to handle and were easily fragmented during solution exchanges. This was observed even after the first 24 h of incubation. At the end of the tenth day, samples subjected to Protocol I (Figures 2B and 3B) became totally translucent and gelatinous, whereas those from Protocol II (Figures 2C and 3C), besides being translucent, showed a significant decrease in size and consistency.

#### Histological analysis

Samples processed according to Protocol I presented remaining cells in both portions of the placenta (Figures 2E and 3E). In HE staining, cell nuclei could be identified mainly in the fetal portion, near the blood vessels (Figure 2E). In the maternal portion, remaining cell nuclei were present in the edges of the samples and were apparently less frequent than in the fetal portion. This demonstrated partial effectiveness of Protocol I (Figure 3E) for decellularization of both portions. Although cell nuclei were not quantified, a decrease of cell number was more evident in placentas subjected to Protocol II; however, this process led to a higher disorganization of the remaining ECM (Figures 2F and 3F). Samples from Protocol I better preserved the ECM architectural structure, including the organization of blood vessels, which was similar to that found in control group. Protocol II promoted greater disarrangement characterized by large spaces between collagen fibers.

Collagen fibers of different diameters were observed under polarized light of sections stained with Picrosirius red (Figures 2J, K, L and 3J, K, L). Images suggest the maintenance of larger collagen fibers (reddish staining) in the fetal portion compared to the maternal portion, where there was predominance of collagen fibers of smaller diameter (greenish staining). Preservation of blood vessel walls, in both portions but particularly in samples processed by Protocol I, was observed (Figures 2H, I and 3H, I).

#### Scanning electron microscopy (SEM)

**Figure 4.**
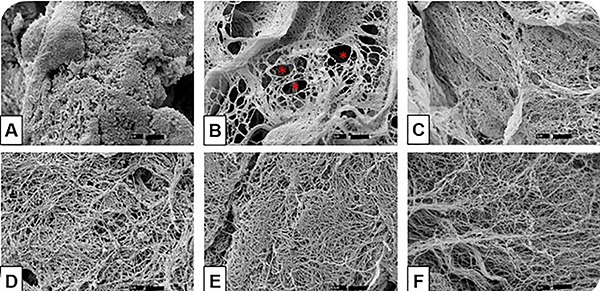
Scanning electron microscopy of fetal (*A–C*) and maternal (*D–F*) portions of canine placenta. *Left column* (control group), *middle column* (Protocol I – 5 mM EDTA + 50 mM TRIS + 0.5% antibiotic) and *right column* (Protocol II – 5 mM EDTA + 0.05% trypsin + 0.5% antibiotic). The red asterisks indicate the spaces that resemble pores observed mainly in fetal portions decellularized with Protocol I (*B*). Scale bar = 3 µm.

SEM analysis (Figure 4) demonstrated the presence of fibrillar components of the ECM, in both placental portions, decellularized by both protocols (Figure 4B, C, E, F). Irregular spaces (resembling pores) formed in between the fibrils were also noticed (Figure 4B).

#### DNA content assay

**Figure 5.**
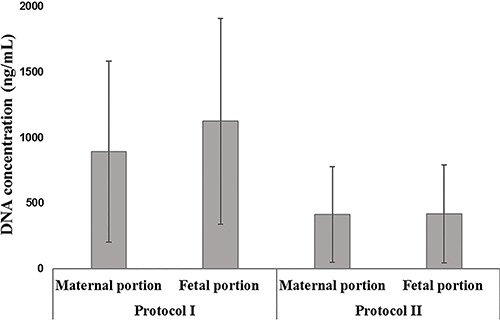
DNA quantification performed using Quant-iTTMPicoGreen^®^dsDNA reagent. Samples subjected to Protocol II (EDTA + 0.05% trypsin + 0.5% antibiotic) showed a trend to less concentration of DNA content compared to samples processed according to Protocol I (5 mM EDTA + 50 mM TRIS + 0.5% antibiotic), but the values were not statistically significant (P>0.05). Data are reported as means±SD (Protocol I: 891±690.7071 for maternal portion and 1125±787.0756 for fetal portion. Protocol II: 412±365.9669 for maternal portion and 418±373.6606 for fetal portion).

The assessment of DNA remaining in the ECM after the decellularization processes showed a trend to less DNA content in the samples processed according to Protocol II, in both maternal (412 ng/mL) and fetal portions (418 ng/mL) compared to Protocol I, which presented DNA concentrations of 891 ng/mL and 1,125 ng/mL, in maternal and fetal portions respectively (Figure 5). However, the difference was not statistically significant when the groups (maternal or fetal portions of Protocols I and II) were compared to each other. Difference was detected (P=0.017) only when Protocol I (the sum of fetal and maternal values) was compared to the combined values observed in Protocol II.

#### Immunofluorescence

**Figure 6.**
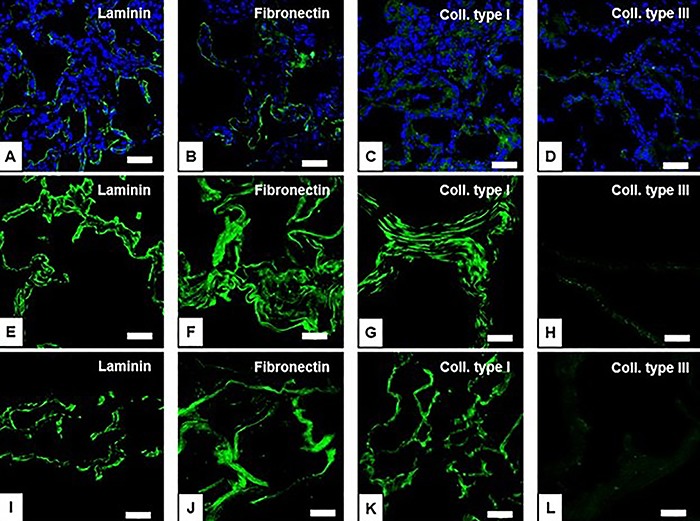
Immunofluorescence of extracellular matrix proteins from fetal portions of canine placenta in control group (*A–D*), Protocol I (5 mM EDTA + 50 mM TRIS + 0.5% antibiotic) (*E–H*), and Protocol II (5 mM EDTA + 0.05% trypsin + 0.5% antibiotic) (*I*–*L*). Laminin, fibronectin, collagen types I and III were expressed in control groups (*A–D*); collagen type III was less evident in the same samples of decellularized groups (*E–L*). Scale bar = 40 µm.

**Figure 7.**
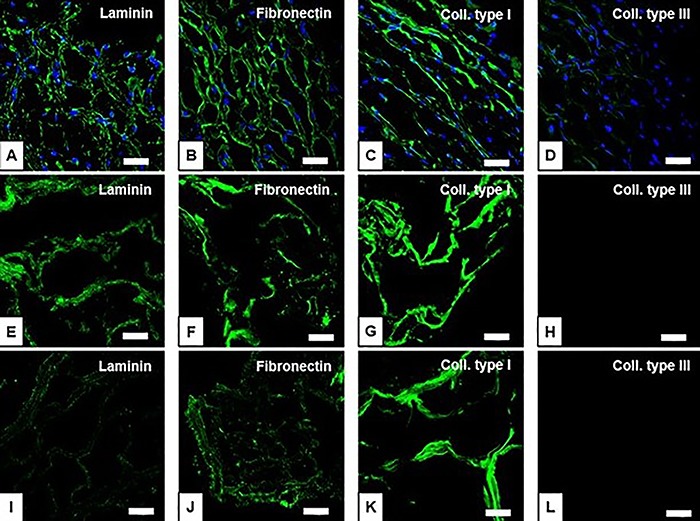
Immunofluorescence of extracellular matrix proteins from maternal portions of canine placenta in control group (*A–D*), Protocol I (5 mM EDTA + 50 mM TRIS + 0.5% antibiotic) (*E–H*), and Protocol II (5 mM EDTA + 0.05% trypsin + 0.5% antibiotic) (*I–L*). Laminin, fibronectin, collagen types I and III were expressed in control groups (*A–D*); collagen type III was less evident (*D*). In decellularized groups, collagen type III was not identified in maternal portions in both protocols (*H, L*). Other proteins were observed in all samples after Protocols I (*E–G*) and II (*I–K*). Nuclear staining with DAPI was absent, indicating cell removal in decellularized groups (*E–L*). Scale bar = 40 µm.

Positive immunostaining was observed for fibronectin, laminin and collagen type I, in both portions of placenta of the control group (Figures 6A-D and 7A-D). Collagen III was not easily detected and was not observed in the maternal portion of decellularized samples, in both protocols (Figure 7H, L). Laminin expression was reduced in the maternal portion of samples decellularized by Protocol II (Figure 7I). Fibronectin was better preserved in the maternal portion of Protocol II (Figure 7J), compared to Protocol I (Figure 7F).

## Discussion

Several decellularization protocols have been applied to different tissues and organs, such as kidney (11), liver (22), heart (23), lung (24), pancreas (25), and to human (12,18,20) and bovine placentas (13). However, the literature lacks information regarding processes for decellularization of canine placentas.

Placentas are heterologous organs with several variations among species and may present large size and thickness. Additionally, they present complex structures, diverse anatomical features and a large vascular network, which suggest that the establishment of a reproducible decellularization protocol may be challenging (12,13).

Combination of decellularization methods (chemical, physical and/or enzymatic) and variables such as perfusion/immersion, duration of incubation and type and concentration of detergents, as well as freezing temperatures used before the beginning of the process, are important parameters that must be evaluated.

Placental shape (girdle in canines) and their vascular tree system are aspects that must be considered for decellularization. After 45 days of gestation, canine placental vascular network is arranged in lobes along the fetal labyrinth zone and establishes connections with adjacent capillary network (26). Solutions perfused through any of these vessels can be distributed throughout this intrinsic vessel network, in the fetal portion of the canine placenta. Therefore, in this study, perfusion with water before the decellularization process was performed to eliminate blood clots as described by Hopper et al. (18) and Choi et al. (19) for perfusion of human placentas.

According to Flynn et al. (12), the combination of several perfusion flows in fetal and maternal placental portions improves the removal of cell content from the ECM. In the present study, the perfusion protocol used (150 mL/h) did not positively impact the decellularization and required greater volume of solutions; therefore, the immersion method was chosen. Other parameters of perfusion should be tested for distinct tissues and/or types of placentas. For instance, Hopper et al. (18), demonstrated that acellular matrices from thicker tissues or with great volume can be difficult to prepare due to resistance in delivering of chemical reagents to the center of the samples. Perfusion can improve distribution of such reagents through all extent of the placentas when a continuous solution flux is established.

Freezing of tissues promotes ice crystal formation within the cell and consequent cellular membrane rupture (27), which may improve cell removal (13). The present investigation showed that freezing canine placentas before the utilization of detergents indeed improved cell removal. However, it required longer time of incubation in detergents to acquire translucency and led to greater disruption of the remaining ECM.

The association of the enzyme trypsin used in Protocol II caused more disorganization of the ECM tridimensional architecture compared to EDTA and TRIS solution, applied in Protocol I. Trypsin disrupts the cell adhesion to the matrix and may lead to indiscriminate destruction of ECM proteins (mainly glycosaminoglycan and elastin), affecting their original conformation. Constant washes to remove this enzymatic solution from the tissues or the utilization of other solutions to inactivate the trypsin action are necessary (1,12,28,29).

Hypo- and hypertonic Tris buffers (30–32) interfere in the relation between DNA and proteins and tend to lyse cells due to osmotic effects. The use of such solutions aims at removing cells (32,33) and cellular remnants (5,7,34).

The ECM disruption of decellularized canine placentas obtained with Protocol II can be due to a relatively large period of time (48 h) of trypsin exposure, in spite of the several washes conducted throughout the process. Lowering the trypsin concentrations may minimize such ECM damages and should be tested in other protocols. The preservation of the tridimensional structure of biological acellular matrices helps the support and development of new tissues (35,36). This, combined with ECM chemical composition, are crucial aspects for the determination of the biological response to organic biomaterials produced by tissue decellularization (36,37).

In this study, the concentration of 1% for the ionic detergent SDS was used based on descriptions in the literature (13). The application of only one type of detergent (SDS or Triton X-100) was inefficient to promote decellularization. Therefore, the association of additional solutions (such as anionic detergent and enzyme solutions) was tested, as described in other decellularization protocols for human placentas (12,18).

Therefore, tissue decellularization commonly leads to acellular matrices with gelatinous and semitransparent aspects and decrease of the tissue length, width and/or thickness (11,28). In addition to these aspects, decellularized canine placentas became more fragile, depending upon the type of detergents and time of incubation used, which can impose some difficulties to handle the future biological acellular biomaterial, at the end of the process. This may ultimately influence the indication of potential clinical applications.

The remaining amount of nucleic acid content in the decellularized ECM is another important aspect that must be evaluated. DNA concentration lower than 50 ng/mg of matrix has been considered necessary to avoid exacerbated inflammatory response after the implantation of acellular matrices. Such exacerbated reaction may compromise recellularization with autologous cells and may lead to rejection. In some cases, immunological responses are not observed immediately, but years after the acellular matrix implantation; therefore, long-term follow-up is necessary (5,8,11). For this reason, the association of DNAse and RNAse is commonly used in decellularization protocols.

The extracellular matrix is composed by glycoproteins, proteoglycans and other proteins, which influence cell behavior, including proliferation and differentiation. The ECM is responsible for maintaining cells and organ function, serving as an intrinsic cellular communication network that provides a supporting structure (7).

The placenta ECM is rich in collagen types I, III, IV, V, and VI, fibronectin and laminin (38). Laminin is a protein of the cell membrane and is commonly observed at basal surface of trophoblast. Its expression increases with advancing gestation (39) and presents correlation with other structural proteins, such as collagen type IV (38). Fibronectin is an abundant protein in placental stroma that is expressed at the basal membrane and walls of blood vessels, in the beginning and in the end of pregnancy (39,40).

Collagen type I, with a diameter between 30-35 nm, is associated with other structural components of the matrix, such as collagen types III, V, VI, and fibronectin. This protein has wide distribution and is observed mainly in villous stroma and walls of blood vessels (38,39). Collagen type III (with diameter ranging between 15-20 nm) is also associated with fibronectin and is frequently interlaced with other collagens fibers, such as collagen type I (38).

These four proteins were found in both portions of canine placenta. Control and decellularized groups were positively stained for collagen type I and fibronectin. Laminin was lightly marked on the maternal portion processed by Protocol II, although its expression was more evident in Protocol I. Collagen type III was not detected after decellularization with Protocols I and II, suggesting that not only the mechanical properties and features but also the chemical composition of the ECM was affected by the process.

One of the main advantages of decellularized matrices for tissue engineering relies on the ECM chemical composition (matrix proteins), which may favor tissue regeneration (19). It is inevitable, however, that the decellularization process, lyophilization and/or sterilization, which are crucial processing steps for further applications, may damage the ECM structure, particularly the protein's folding, which tends to directly influence tissue response (35).

Although collagen type III was eliminated from the maternal portion in both protocols analyzed in this study, others matrix proteins (laminin, fibronectin and collagen type I) were preserved. These components, along with growth factors, have been shown to be important, especially in cases of skin wound healing (18).

In conclusion, protocols for decellularization are tissue-dependent. The detergent SDS is crucial but insufficient to promote cell removal. The association of the enzyme trypsin (Protocol II) favored cell removal but decreased tissue integrity compared to the buffer Tris (Protocol I). Lowering the DNA content towards acceptable established parameters is essential and may influence biocompatibility and biosafety of biological acellular matrices. Alterations of the composition and bioactivity of the remaining proteins, together with the final size, shape and handling properties of the acellular matrix are also aspects to be considered when selecting a protocol for tissue decellularization.
